# DMF inhibits PDGF-BB induced airway smooth muscle cell proliferation through induction of heme-oxygenase-1

**DOI:** 10.1186/1465-9921-11-145

**Published:** 2010-10-20

**Authors:** Petra Seidel, Stephanie Goulet, Katrin Hostettler, Michael Tamm, Michael Roth

**Affiliations:** 1 Pulmonary Cell Research, Department of Biomedicine and Pneumology, Department of Internal Medicine, University Hospital Basel, Switzerland

## Abstract

**Background:**

Airway wall remodelling is an important pathology of asthma. Growth factor induced airway smooth muscle cell (ASMC) proliferation is thought to be the major cause of airway wall thickening in asthma. Earlier we reported that Dimethylfumarate (DMF) inhibits platelet-derived growth factor (PDGF)-BB induced mitogen and stress activated kinase (MSK)-1 and CREB activity as well as IL-6 secretion by ASMC. In addition, DMF altered intracellular glutathione levels and thereby reduced proliferation of other cell types.

**Methods:**

We investigated the effect of DMF on PDGF-BB induced ASMC proliferation, on mitogen activated protein kinase (MAPK) activation; and on heme oxygenase (HO)-1 expression. ASMC were pre-incubated for 1 hour with DMF and/or glutathione ethylester (GSH-OEt), SB203580, hemin, cobalt-protoporphyrin (CoPP), or siRNA specific to HO-1 before stimulation with PDGF-BB (10 ng/ml).

**Results:**

PDGF-BB induced ASMC proliferation was inhibited in a dose-dependant manner by DMF. PDGF-BB induced the phosphorylation of ERK1/2 and p38 MAPK, but not of JNK. DMF enhanced the PDGF-BB induced phosphorylation of p38 MAPK and there by up-regulated the expression of HO-1. HO-1 induction inhibited the proliferative effect of PDGF-BB. HO-1 expression was reversed by GSH-OEt, or p38 MAPK inhibition, or HO-1 siRNA, which all reversed the anti-proliferative effect of DMF.

**Conclusion:**

Our data indicate that DMF inhibits ASMC proliferation by reducing the intracellular GSH level with subsequent activation of p38 MAPK and induction of HO-1. Thus, DMF might reduce ASMC and airway remodelling processes in asthma.

## Background

Asthma is a chronic inflammatory disease of the airways that is characterised by airway hyper-responsiveness (AHR), increased broncho-constriction, and an increased airway wall thickness [1]. The increase of the airway smooth muscle cell (ASMC) mass in asthma results in thickening of the airway wall by increasing the mass of contractile cells and reduction of the bronchial lumen. Increased levels of platelet-derived growth factor (PDGF)-BB have been reported in asthma patients' lung and may contribute to the increased ASMC mass [2-6].

Inhaled glucocorticoids (GC) remain the most effective anti-inflammatory therapy in chronic lung diseases [7]. In an earlier study, we and others showed that glucocorticoids may have no anti-proliferative effect on ASMC of asthma patients due to a deficiency in C/EBP-α [8] which is essential to form a complex with the activated glucocorticoid receptor in order to induce p21 [9,10]. In a recent publication, the lack of the anti-proliferative effect of glucocorticoids on asthmatic ASMC was confirmed. Interestingly, vitamin D acted as an anti-proliferative agent further down-stream of p21^(waf1/cip1)^, namely on p53 [11].

Fumaric acid esters (FAE) including dimethylfumarate (DMF) are registered in Germany for the therapy of severe psoriasis. Furthermore, the clinical efficacy of DMF to reduce inflammation in multiples sclerosis has been demonstrated [12]. Some of psoriasis and multiples sclerosis patients who also suffered from asthma reported that DMF reduced their asthma symptoms and improved the overall quality of life.

Focusing on the anti-proliferative properties of DMF, several mechanisms have been described by which DMF can achieve this effect. In human colon carcinoma cells, DMF inhibited cell proliferation by down regulating intracellular GSH levels [13]. Similarly, in human T-lymphmphocytes, the DMF reduced cell proliferation was rescued by exogeneous glutathione [14]. Other *in vitro *studies showed that DMF down-regulated the level of cellular glutathione (GSH) in epithelial cells and asterocytes [15,16]. In human lung fibroblasts, depletion of GSH up-regulates the enzyme heme oxygenase (HO)-1 [17]. Interestingly in human ASMC, HO-1 inhibited cell proliferation [18]. Earlier we have reported that DMF inhibited PDGF-BB induced MSK-1 and CREB activation, thereby down-regulating the secretion of IL-6, eotaxin and RANTES [19]. However, it remains unknown whether DMF has anti-proliferative properties in ASMC.

In this study we determined the effect of DMF on PDGF-BB induced ASMC proliferation and HO-1 expression. In addition, we assessed the drugs effect on intracellular GSH and its role in proliferation control. Furthermore, we determined the role of JNK, p38, and ERK1/2 MAPK activation and GSH in DMF induced HO-1 expression.

## Methods

### Isolation, characterisation and culture of human ASMC

**Figure 1:**
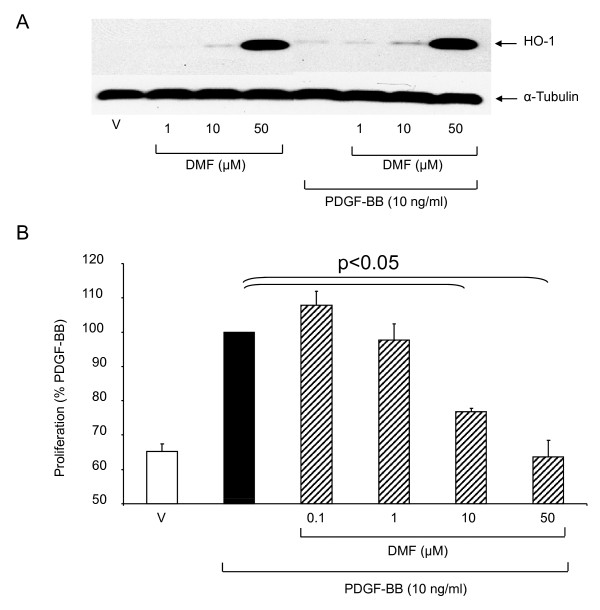
**DMF induces heme-oxygenase-1 (HO-1) expression and inhibits proliferation in primary ASMC. **(A) a representative immuno-blot of the concentration-dependent effect of DMF on HO-1 expression at 24 h by ASMC. Similar results were obtained in three cell lines. "V" indicates the drug's vehicle 0.05% DMSO. (B) DMF inhibited PDGF-BB induced fibroblast proliferation (24 h). Data represents the mean ± SEM of six independent experiments performed in 3 ASMC lines. Statistics have been calculated by Mann Whitney test. "V" indicates the drug's vehicle 0.05% DMSO.

Human ASMC were isolated and grown from bronchi of resected unused lung tissue obtained from transplant donors as previously described [20]. ASMC were grown in RPMI-1640 (ThermoTrace, Melbourne, Australia) supplemented with 5% (v/v) heat-inactivated fetal bovine serum (FBS), 1× (v/v) MEM vitamin-mix, 100 U/l penicillin, 100 μg/ml streptomycin, and 0.25 μg/ml of amphotericin B (all: GIBCO/BRL, Melbourne, Australia), 25 mM HEPES and 2 mM L-glutamine (ThermoTrace) in a humidified atmosphere at 37°C, in 5% CO_2_, and 95% air (v/v). All ASMC lines were used between passages 5-8. ASMC were characterised by positive immuno-staining for α-SMA and calponin [20] as shown in Figure 1A.

### Drug preparation

DMF (0.1-50 μM), SB203580 (10 μM), Hemin (1-10 μM), and cobalt-protoporphyrin (2-20 μM) were dissolved in dimethysulfoxide (DMSO; all Sigma, Buchs, Switzerland) and diluted to the required concentration in serum free medium. Glutathione-ethylester (GSH-OEt, 1 mM, Sigma) was dissolved in serum free medium.

### HO-1 suppression by siRNA

HO-1 expression in subconfluent cells was down regulated while the cells where serum deprived for 24 hours by transfecting the cells with HO-1 siRNA (Santa Cruz Biotechnology, Santa Cruz, USA: cat# sc-35554) as described previously (19). After 24 hours cells were stimulated with PDGF-BB (10 ng/ml) and/or DMF (1 or 10 μM) and proliferation was determined after 72 hours by direct cell counts.

### HO-1 expression and MAPK (p38, JNK, ERK 1/2) activation

ASMC were grown in 6-well plates to confluence and were then deprived of serum for 24 hours. The cells were then pre-treated for 1 hour with a single drug, or with a drug combination, before being stimulated with PDGF-BB (10 ng/ml). Total cell lysates were collected at 0, 5, 10, 15, 30 or 60 minutes and MAPK expression and activation was determined by immuno-blot. HO-1 expression was determined at 24 hours.

### Immunoblotting

Protein extracts were size-fractionated by SDS-PAGE electrophoresis and transferred onto nitrocellulose membranes as described previously [19]. Protein transfer was confirmed by Ponceau staining. Membranes were incubated with blocking buffer (5% w/v non-fat dry milk in Tris-buffered saline containing 0.1% Tween 20) for 1 h at room temperature and were then incubated with one of the following primary antibodies: anti-p38, anti-phospho-p38, anti-ERK1/2, anti-phospho-ERK1/2, anti-JNK, anti-phospho-JNK (all Cell Signalling Technology, Beverly, MA), anti-HO-1 (Calbiochem, Luzern, Switzerland) anti-α-Tubulin (Santa Cruz, Santa Cruz, USA). Primary antibodies were detected by horseradish peroxidase-conjugated IgG antibodies diluted 1:2000-1:40000 (anti-rabbit IgG sc-2004, or anti-mouse IgG sc2005; Santa Cruz) and protein bands were visualised by enhanced chemiluminescence (Pierce Biotechnology Inc. Rockford, USA).

### ASMC proliferation

Proliferation was measured by [^3^H]-thymidine incorporation. In brief, ASMC were seeded into 96-well plates at 60% confluence and serum-deprived for 24 hours. Cells were pre-treated for 1 h with the different drugs alone or in combination and stimulated with PDGF-BB (10 ng/ml) for 24 hours. During the final 3 hours, 2 μCi/ml [^3^H]-thymidine (Amersham) were added and the incorporated [^3^H]-thymidine was determined by liquid scintillation counting [21].

In addition, manual cell counts were performed after 3 days of culture as described earlier using an improved Neugbaur chamber slide [20]. Subconfluent ASMC were transfected with HO-1 siRNA as previously described above and then deprived of serum for 24 hours. ASMC were then treated with DMF (1, 10 μM) and/or PDGF-BB (10 ng/ml) and proliferation was determined by manual cell counts after 72 hours.

### Data analysis

Proliferation data are expressed as mean ± S.E.M. The statistical analysis was performed using the two-sided Wilcoxon-Mann-Whitney U-test.

## Results and Discussion

In this study we show that DMF inhibits the pro-proliferative action of the asthma relevant growth factor PDGF-BB on human ASMC via an increase of HO-1. DMF achieves this effect by depletion of intracellular GSH which in turn activates p38 MAPK by a yet un-known mechanism and this increases the expression of HO-1.

In asthma characteristic structural abnormalities of the airway wall include an excessive accumulation of ASMC which express increased levels of connective tissue elements. Importantly, these pathologies correlated with severity of the disease and were widely resistant to conventional therapies [22]. Further evidence for the significant contribution of ASMC to asthma comes from a novel form of therapy: thermoplasty of ASMC. This therapy eliminated ASMC by over heating the cells by means of radio-waves and results in lasting improvement of severe asthma [23]. Besides remodelling, ASMC actively sustain or increase inflammation in asthma by secreting a large range of pro-inflammatory cytokines and various pro-inflammatory components of the extracellular matrix [24,25]. Especially the asthma relevant growth factor PDGF-BB induced ASMC proliferation, as well as it activated the cells to secrete components of the extracellular matrix [25,26]. Thus, reducing the number of ASMC in asthma may resolve several pathologies and therefore improve lung function and quality of life.

In ASMC treated with the vehicle in the presence or absence of PDGF-BB, we observed no induction of HO-1 expression as shown in a representative immuno-blot in Figure 1A. In contrast, DMF induced the expression of HO-1 in a dose-dependent manner and this effect was not altered by the presence of PDGF-BB (Figure 1B). The PDGF-BB induced the proliferation of ASMC was significantly inhibited in a dose-dependent manner by DMF at concentration >1 μM (Figure 1B).

**Figure 2:**
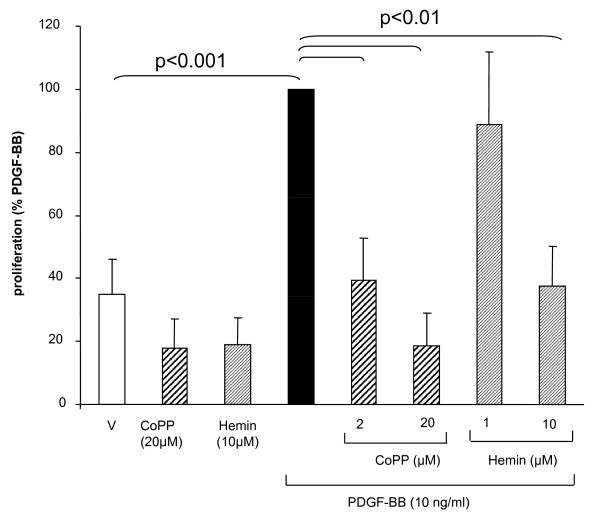
**HO-1 induction inhibits ASMC proliferation. **The HO-1 inducer cobalt-protoporphyrin (CoPP) and hemin inhibited PDGF-BB induced fibroblast proliferation after 24 h. Data represents mean ± SEM of six independent experiments performed in 3 ASMC lines. Statistics have been calculated by Mann Whitney test. "V" indicates the drug's vehicle 0.05% DMSO.

Together with others our observation suggests that DMF is a strong inducer of HO-1 in smooth muscle cells, as the drug inhibited remodelling of pulmonary vessels in rats, and thus indicates an overall anti-remodelling effect [27]. Furthermore, HO-1 expression reduced hypoxia induced pulmonary vessel remodelling in rats with chronic pulmonary heart disease [28]. In order to determine whether the induction of HO-1 mediates the anti-proliferative effect of DMF in ASMC, we treated the cells with the HO-1 inducers hemin (1-10 μM) or cobalt-protoporphyrin (2-20 μM) 1 hour before stimulation with PDGF-BB. As depicted in Figure 2, Cobalt-protoporphyrin dose-dependently reduced PDGF-BB induced ASMC proliferation. Hemin however, showed such an effect only at the highest concentration (10 μM) (Figure 2). Neither Cobalt-protoporphyrin (20 μM) nor hemin (10 μM) had any significant effect on ASMC proliferation in un-stimulated cells (Figure 2). Our finding that other HO-1 inducers (hemin, CoPP) also inhibit cell proliferation of is in agreement with reports that HO-1 reduced the proliferation of isolated human lymphocytes [14] and of pancreatic stellate cells [29].

**Figure 3:**
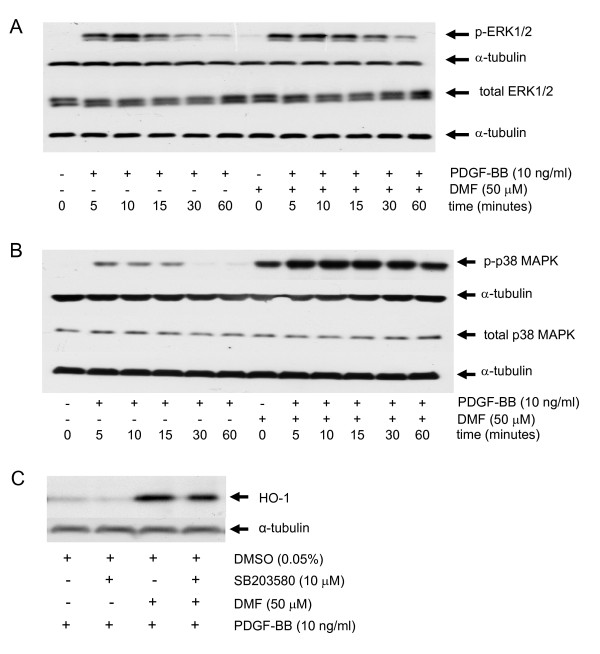
**DMF activates phosphorylation of p38 MAP kinase and p38 MAPK inhibition reduces DMF induced HO-1 in ASMC. **(A) a representative immuno-blot of the PDGF-BB induced ERK1/2 MAPK (p-ERK1/2) phosphorylation kinetic in the presence and absence of DMF. Similar results were obtained in three additional cell lines. (B) a representative immuno-blot of the kinetic of PDGF-BB induced ERK1/2 MAPK (p-ERK1/2) phosphorylation and its enhancement by DMF; similar results were obtained in three cell lines. (C) a representative immuno-blot of DMF-induced HO-1 expression and its reduction by the p38 MAPK inhibitor SB203580 Similar results were obtained in three cell lines.

Furthermore, in line with our results, GSH depletion was reported to increase the phosphorylation of p38 MAPK in C6 glioma cells [30]. Taken together with the observation that HO-1 is regulated by p38 MAPK [29] our data suggest that DMF and GSH reduction augment HO-1 expression in a p38 MAPK dependent way. In our experimental conditions PDGF-BB strongly activated the phosphorylation of ERK 1/2 MAPK between 5-30 minutes, and DMF had no effect on this signalling activity (Figure 3A). PDGF-BB induced the phosphorylation of p38 MAPK within 5-15 minutes, declining to baseline levels thereafter (Figure 3B). Surprisingly, the pre-incubation of ASMC with DMF activated p38 MAPK significantly (Figure 3B, lane 7) and the combination of PDGF-BB with DMF (50 μM) further increased and prolonged p38 MAPK activity (Figure 3B). Neither PDGF-BB, nor DMF alone or in combination had any effect on the expression of total p38, or ERK 1/2 MAPK (Figure 3A,B). JNK phosphorylation was neither induced by PDGF-BB nor by DMF.

Next we assessed the possible link between DMF induced activation of p38 MAPK and the induction of HO-1 expression. As shown in Figure 3C, the p38 MAPK inhibitor SB203580 partly reversed the DMF induced expression of HO-1, whereas SB203580 alone had no effect on HO-1 level.

**Figure 4:**
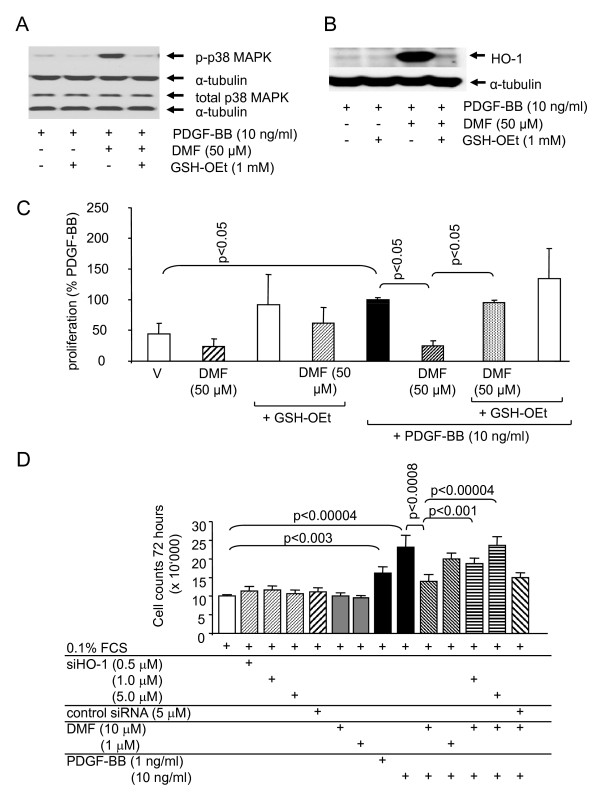
**GSH reverses the effects of DMF on p38 MAPK phosphorylation, on HO-1 expression and on ASMC proliferation. **(A) a representative immuno-blots of the reversing effect of GSH on DMF- and PDGF-BB induced of p38 MAPK phosphorylation in ASMC at 30 min. Similar results were obtained in three cell lines.. (B) a representative immuno-blot of the reversing effect of GSH on the DMF-induced HO-1 expression at 24 h; similar results were obtained in three additional cell lines.. (C) a counteractive effect of GSH on DMF dependent inhibition of ASMC proliferation. Similar results were obtained in four cell lines. Data represents mean ± SEM (unpaired student's t-test). "V" indicates the drug's vehicle 0.05% DMSO. (D) down regulation of HO-1 by a respective siRNAs counteracted the anti-proliferative effect of DMF. Data represents the mean ± SEM of 9 independent experiments performed in 3 ASMC lines. Statistics have been calculated by Wilcoxon-Mann-Whitney U-test.

A role of oxidative stress, GSH and of its major metabolizing enzyme glutathione-S transferase in asthma has been proposed [31], but the data was led to controversial interpretations and the underlying mechanism is not understood [32,33]. Like other studies, we observed that the anti-proliferative action of DMF was linked to its ability to reduce intracellular GSH level [13,15,16]. The reduction of intracellular GSH increased the expression of HO-1 by human lung fibroblasts [34], and the depletion of GSH in airway epithelial cells ASMC up-regulated HO-1 expression [35,36]. These studies support our finding that DMF inhibits ASMC proliferation by depletion of GSH and up-regulating of HO-1. As shown above, DMF enhanced PDGF-BB induced p38 MAPK phosphorylation and this effect was completely reversed in the presence of GSH-OEt (Figure 4A). GSH-OEt itself had no significant effect on PDGF-BB induced p38 MAPK activation (Figure 4A). The DMF-induced expression of HO-1 was completely suppressed in the presence of GSH, whereas GSH-OEt alone had no effect (Figure 4B).

As shown in figure 4C, neither the drug vehicle (DMSO) nor DMF alone had significant effects on non-stimulated ASMC proliferation (bars 1, 2). GSH-OEt increased proliferation under all conditions, but this effect did not become significant compared to the respective controls (bars 3, 4, 8). Most importantly, the PDGF-BB induced ASMC proliferation (bar 5) was significantly inhibited by 1 hour pre-incubation with DMF (bar 6) and this effect was reversed by the addition of GSH-OEt (bar 7).

We further investigated the role of HO-1 in DMF dependent inhibition of cell proliferation by the use of various concentration of siRNA targeting HO-1 as shown in figure 4D. HO-1 siRNA alone did not significantly change cell proliferation in serum deprived ASMC over 3 days (Figure 4D). Similarly, the control siRNA or DMF (10^-6 ^- 10^-5^M). PDGF-BB dose dependently increased cell numbers by maximal 2.4 folds over 3 days and this effect was dose dependently inhibited by DMF (Figure 4D). The pre-incubation of ASMC with HO-1 siRNA, prior to the addition of PDGF-BB and DMF, counteracted the anti-proliferative effect of DMF in a clearly dose dependent pattern as shown in figure 4D. This finding supports the hypothesis that HO-1 mediates the anti-proliferative effect of DMF.

## Conclusions

In conclusion, our data show that DMF down-regulates PDGF-BB induced proliferation of ASMC through a GSH and p38 MAPK dependent induction of HO-1. The clinical efficacy of DMF and its safety profile in psoriasis and multiples sclerosis makes it an interesting drug that may help to reduce airway wall remodelling and inflammation in chronic inflammatory lung diseases such as asthma and COPD.

## Competing interests

The authors declare that they have no competing interests.

## Authors' contributions

PS has contributed to the study design, cell culture, immuno-blotting, data analysis and manuscript preparation. SG has contributed to the work in cells proliferation and drugs testing. KH has contributed to the cell culture and siRNA experiments. MT has contributed to the study design. MR has contributed to the study design, cell culture, data analysis as well as manuscript preparation. All authors read and approved the final manuscript.
